# The impact of The Quality and Safety Education (QSEN) program on the knowledge, skills, and attitudes of junior nurses

**DOI:** 10.1371/journal.pone.0317448

**Published:** 2025-01-24

**Authors:** Salam AlRatrout, Imad Abu Khader, Mohammed ALBashtawy, Mohammed Asia, Abdullah Alkhawaldeh, Salam Bani Hani

**Affiliations:** 1 Palestinian Ministry of Health, Palestine; 2 Assistant of Vice President for Medical Faculties Affairs, Arab American University, Jenin, Palestine; 3 Department of Community and Mental Health Nursing, Princess Salma Faculty of Nursing, Al al-Bayt University, Mafraq, Jordan; 4 Vice President for Medical Faculties ‘Affairs, The Arab American University-Jenin, Jenin, West Bank, Palestine; 5 Nursing Department, Faculty of Nursing, Irbid National University, Jordan, Irbid; University of Hafr Al-Batin, SAUDI ARABIA

## Abstract

**Background:**

The quality and safety education for nurses (QSEN) competency program represents a valuable initiative in nursing practice and education, equipping nurses with the essential knowledge, attitude, and skills (KAS) required to deliver safe, efficient, and patient-centered care.

**Purpose:**

This study aims to determine the impact of QSEN competency on the KAS of nurses in Palestine.

**Method:**

A quasi-experimental pre-test and post-test design with two groups was used utilizing a questionnaire to collect data from 164 Junior nurses in two governmental hospitals within the period of 25^th^, January to the 10^th^ February 2024. Patricia Benner’s theory suggests that a strong educational foundation and diverse experiences enable nurses to enhance their patient care knowledge and abilities over time.

**Results:**

The findings indicate that nurses in Palestine can benefit from targeted interventions and QSEN educational programs aimed at improving their patient-centered care competence, as post-test scores show a significant rise over pre-test scores. Junior nurses who participated in the QSEN program experienced a 57% increase in knowledge, a 57% increase in skills, and a 64% increase in attitudes. The intervention significantly improved knowledge (77.02 vs. 49.19, p < 0.001), quality and safety skills (70.16 vs. 44.61, p < 0.001), and attitudes (75.47 vs. 46.16, p < 0.001) among participants post-procedure, indicating a substantial positive impact on these areas, demonstrating the effectiveness of the educational intervention.

**Conclusion:**

The study demonstrates that an educational intervention improves junior nurses’ KSAs for six QSEN competencies, leading to higher average scores in quality and safety competence subscales, thereby enhancing staff satisfaction, and reducing medical errors, and patient safety.

## Introduction

Patient Safety (PS) is a major concern in the healthcare industry, where it is estimated that 43 million safety incidents occur annually [1]. This multifaceted concept includes a variety of actions meant to reduce risks associated with providing healthcare, promote a strong culture of safety, and protect patients from harm to ensure their well-being as well as a surveillance system [2].

A safety-conscious culture is a crucial component in healthcare systems, ensuring patient safety and reducing hazards [3]. This approach has gained global recognition, emphasizing the need for efficient strategies to enhance healthcare systems’ safety [2]. Globally, PS remains a paramount concern across all healthcare systems. According to the WHO, there was a great emphasis on the importance of PS in ensuring safe and error-free healthcare, emphasizing the role of nurses as leaders in safeguarding this crucial aspect [4].

Across the world, patient care systems’ safety and quality are major problems [5]. These worries are made worse in the Palestinian Ministry of Health by issues like a high patient-to-nurse ratio, scarce resources, and unstable political environments. Junior nurses might not have the thorough information, abilities, and attitudes required to guarantee excellent and secure patient care because they are frequently recent graduates with less experience [6]. This lack of readiness may result in worse-than-ideal patient outcomes, a rise in medical mistakes, and general inefficiencies within the healthcare system.

The Quality and Safety Education for Nurses (QSEN) initiative aims to prepare future nurses with the knowledge, skills, and attitudes necessary to improve healthcare systems’ quality and safety continuously. QSEN competencies have been integrated into nursing education in various countries, adapting to their unique healthcare contexts and educational frameworks [7]. For instance, in the United States, the QSEN competencies are widely integrated into nursing education programs such as The American Association of Colleges of Nursing (AACN) and the National League for Nursing (NLN), also these competencies are included in accreditation standards and licensure examinations [8]. In Canada, there was a Canadian Association of Schools of Nursing (CASN) that emphasizes competencies similar to QSEN through the incorporation of quality and safety into the national nursing curriculum [9]. While in the Middle East, in Jordan great efforts were being made to integrate QSEN competencies into nursing education, with an emphasis on PCC and teamwork. This reflects a broader trend in the region to improve healthcare quality and safety [10].

QSEN competency programs are crucial in providing nurses with the core skills required to deliver Patient-Centered Care (PCC) that is safe, efficient, and compliant. The QSEN program is intended for junior nurses who work for the Palestinian Ministry of Health [11]. These nurses may have just finished their nursing school and are often in the beginning phases of their profession. These initiatives help nurses provide more individualized care that respects each patient’s needs and preferences, which improves patient outcomes and raises the standard of healthcare as a whole [12]. Junior nurses have special needs since they often struggle to apply theoretical knowledge in practical settings and their hands-on training emphasizes real-world application of theoretical concepts, including simulation-based learning and supervised clinical practice. Fostering a culture of safety in healthcare facilities is accelerated by the inclusion of QSEN competencies in nursing programs [13]. It eventually ensures the provision of safe and excellent care by placing a strong emphasis on fostering collaboration and teamwork among medical professionals and developing a feeling of shared responsibility for PS. In addition, QSEN competency programs give nurses the chance to actively participate in evidenced-based practices (EBP), informatics, and Quality Improvement (QI) projects—all of which are meant to improve PS and healthcare outcomes [14].

Junior nurses, as (primary healthcare) PHC providers play a crucial role in delivering high-quality care [15]. However, nurses face challenges in implementing evidence-based procedures due to a lack of comprehensive quality and safety instruction in nursing programs [16]. The WHO highlights the need for a QSEN competency-based program to improve PS outcomes and healthcare quality, highlighting the need for empirical evidence [2]. The current literature provides insufficient details regarding the influence of the QSEN competency program on junior nurses’ KAS.

As a result, empirical data is needed to bridge this knowledge gap and add to the body of knowledge already in existence. The study intends to enhance the general quality and safety of patient care by providing junior nurses with QSEN competencies. This will improve patient outcomes and lower the rate of medical errors.

The geographical and sociopolitical background of this study is distinct. Palestine’s healthcare system suffers several obstacles, such as a lack of resources and unstable political conditions. Evaluating QSEN’s effect in this particular circumstance yields insightful information that may be applied to other comparable situations. Even while QSEN programs have been extensively researched in a variety of settings, concentrating on junior nurses just starting in their careers can offer crucial perspectives on how early intervention affects their practice and professional growth. Hence, this study aims to determine the impact of the QSEN competency education program on junior nurses’ knowledge, attitudes, and skills toward patient care in the Palestinian Ministry of Health. The QSEN-based program is hypothized to dramatically improve the knowledge, attitudes, and abilities of junior nurses at the Palestinian Ministry of Health.

This study will address the following research questions:

1. How does the QSEN program influence junior nurses’ knowledge, attitude, and skills of patient safety principles in clinical practice?
2. What is the impact of the QSEN program on junior nurses’ knowledge, attitude and skills of evidence-based practice in Palestine?

### Theoretical framework

This study is based on Patricia Benner’s theory [17] which explains how a solid educational foundation and a variety of experiences help nurses grow their abilities and knowledge of patient care over time. The Dreyfus Model of Skill Acquisition, which describes five phases of proficiency—novice, advanced beginner, competent, proficient, and expert—lays the foundation for this idea. According to Patricia Benner’s perspective, nurses gain knowledge and comprehension of patient care through education and experience over time. Five levels of nursing skill are distinguished by this classification: competent, proficient, expert, advanced beginning, and novice which are progressed gradually among junior nurses in Palestine Ministry of Health hospitals. The effort can effectively assist junior nurses in their transition from novices to experts by matching the QSEN-based curriculum with Benner’s Novice to novice-to-expert theory. This strategy makes sure they acquire the know-how, abilities, and mindset needed to deliver safe, effective patient care while taking into account the particular difficulties presented by the Palestinian healthcare system.

## Materials and method

### Study design

A quasi-experimental within and between-subject design was used. Quasi-experimental designs may enhance the external validity of the study by reflecting real-world conditions more closely. This is important for translating research findings into practical applications in healthcare. The study adhered to the guidelines provided in the TREND Statement checklist.

### Study settings and population

The research was conducted in two governmental hospitals. Both of the selected hospitals were educational-affiliated hospitals and are a reference for the Palestinian MOH and their diverse departments covering a range of medical specialties. These two hospitals are The PMC Hospital in Ramallah and Rafidia Hospital in Nablus.

**PMC**, originally named the Ramallah Governmental Hospital, is a strategically important healthcare institution in the central region of the West Bank. It includes the Bahraini Hospital for Children, the Kuwaiti Hospital for Heart and Specialized Surgeries, and the Sheikh Zayed Hospital for Emergency Care. PMC serves as a reference and teaching hospital with various departments such as pediatrics, emergency medicine, internal medicine, general surgery, orthopedics, critical care, and gynecology. The hospital has 490 beds, around 1,050 administrative and medical staff, and a dedicated nursing team of 400. **Rafidia Hospital**, located in Nablus City, is a governmental surgical facility founded in 1976. As one of the largest healthcare institutions in the northern region of the West Bank. It has a bed capacity of 207, and the hospital serves as a crucial healthcare center, providing specialized surgical treatments [18].

### Sampling

The study examined registered nurses currently working in different departments at PMC in Ramallah and Rafidia hospitals in Nablus. The sample included a total of 164 nurses, with 100 from PMC and 64 from Rafidia Hospital. The study utilized G*Power to determine the required sample size, guided by a medium effect size of 0.5 with a significance level α of 0.05 and a statistical power of 0.8 [19]. A total of 64 participants per group were calculated as necessary for conducting a t-test to detect the expected difference between groups. Researchers employed a random sampling method to select participants based on pre-defined inclusion criteria from two hospitals. They divided the selected participants into two groups: an intervention group from PMC hospital that received the QSEN competency program, and a control group from Rafidia hospital that did not receive the program. A comprehensive list of participants was created, including PMC Nurses (N1, N2, N100) and Rafidia Nurses (N1, N2, N64), totaling 164 participants. Sixty-four participants from Rafidia Hospital were purposefully chosen for the control group, while the remaining 100 participants were selected for the intervention group, all based on predetermined inclusion criteria. Participants are required to be (a) registered nurses including five years of experience or less. (b) work in one of the following departments: internal medicine, surgical, pediatric, intensive care unit (ICU), daily care, and neurosurgery departments. On the other hand, nurses with more than five years of practical experience, midwives, and nurses in other departments were not included in the study.

### Data source and measurement

The QSEN competency program is a complete method of nursing education and practice that gives nurses the KSAs required to collaborate with interdisciplinary teams to deliver safe, high-quality, PCC [8]. It places a focus on constant learning, advancement, and ethical issues in healthcare. By putting QSEN competency into practice in a hospital setting, nurses are given the tools they need to deliver PCC effectively to enhance healthcare outcomes and foster a culture of ongoing learning and QI throughout the healthcare system [20]. The researcher’s goal is to provide information on practical methods for raising nurses’ KSAs, which will subsequently improve PS and healthcare standards. The following teaching methods were employed including, Lectures; Case-Based Learning; Simulation: Group discussions; Role-playing; Problem-based learning; Flipped classroom; and Experiential Learning.

To raise PS and the caliber of healthcare, researchers have used a training tool that is specifically designed for QSEN Competency. This tool gives junior nurses the KSAs they need to provide safe and effective care by utilizing visual aids, videos, interactive software, checklists, patient stories, models and displays, and demonstrations.

The context of QSEN competency involves evaluating whether nurses have acquired the KSAs necessary for safe and high-quality patient care through utilizing the following methods, clearly defining learning outcomes, using diverse assessment methods, direct observation, written assessments, and skills assessment.

A self-completion questionnaire was developed to collect information on the participants’ demographic data as well as their QSEN competency results from the program. It included a variety of questions to assess nurses’ competencies in a range of important areas, including patient-centered care, Teamwork, EBP, QI, Safety, and Informatics, and also to assess nurses’ KSAs regarding patient-centered care. The questionnaire consisted of structured questions with defined response formats, making it easier for participants to complete. The questionnaire was given to both the intervention group, which received the QSEN competency program, and the control group, which did not get the program. Through a comparative analysis of responses from the two distinct groups, the efficacy of the QSEN competency program was systematically evaluated. The assessment of the QSEN competency program’s potential impact was realized through the administration of a structured questionnaire to the nursing participants on two occasions first, before the initiation of the intervention program, and subsequently after its completion.

The study utilized a self-administered questionnaire that contains three parts, **Part I (Social Demographics)** includes six questions: The nurse’s age, sex, marital status, higher education (master’s degree or high diploma), years of experience, and name of the university graduated from. **Part II: Clinical Performance Evaluation Questionnaire (CPEQ)** that originated [21]. The CPEQ is a tool used to measure the clinical performance of healthcare professionals. It is a self-administered questionnaire comprising 32 items divided into six domains, including PCC items 8 items, teamwork 7 items, EBP 2 items 3 items, safety 6 items, and informatics 6 items. The internal consistency measured by Cronbach’s alpha of 0.70 indicates good internal consistency, test-retest correlations varied from 0.57 to 0.85. There were 127 items in the completed survey. Five-point ratings on items about care experiences ranged from "not at all" (1) to "a very large extent" (5). Reverse coding was used to identify negative elements, and a higher score indicated a better experience overall. Scales were converted to scores, with 100 representing the best outcome, on a scale of 0 to 100. **Part three: Patient-centered Care Scale (KSAI-PCCS)** that was developed by Esslin [22]. The KSAI-PCCS is a comprehensive tool designed to assess nurses’ patient-centered KSAs. The KSAI-PCCS is a 54-item instrument with three subscales: Knowledge (19 items), Skills (17 items), and Attitudes (18 items)—KSA. The instrument subjectively measures the three domains of PCC competencies for nursing practice. On a scale of 0 to 5 rate the frequency of ability and application of knowledge, skills, and attitudes specific to patient-centered care, where 0 = never, 1 = very rarely, 2 = rarely, 3 = occasionally, 4 = frequently, and 5 = very frequently. Cronbach’s alpha for each of the KSA subscales was greater than .70 with a minimum score of 10 and a maximum of 50. The higher score indicates strong knowledge, well-developed skills, and positive attitudes toward patient-centered care and vice versa.

The QSEN competency program serves as a key component of the research study. This tool is designed to implement the QSEN competency program, which focuses on enhancing the knowledge and skills of nurses in the areas of quality and safety in healthcare. The tool includes structured interventions, educational resources, and training modules aimed at improving nurses’ abilities to provide high-quality, safe patient care. Through the QSEN competency program, participants receive targeted education and training, ultimately enhancing their competence in these critical areas. This intervention tool assesses the impact of the QSEN competency program on nurses’ performance and their ability to deliver quality and safe healthcare services. The QSEN competency initiative is a national initiative that aims to transform nursing education by integrating six core competencies: PCC, Teamwork and Collaboration, EBP, QI, Safety, and Informatics.

This QSEN competency program was extracted from QSEN competency institutions, the WHO PS Assessment Manual, and the literary work authored by Christie in 2014, titled "Introduction to QSEN, Core Competencies, intricately strives to elevate the standards of nursing education and, concomitantly, enhance patient care. The QSEN competency program modules created by the QSEN competency institutions aim to enhance nursing education and patient care by providing a comprehensive understanding of quality and safety in healthcare. The modules cover essential topics, including the critical role of nurses, the imperative of quality and safety, and the enhancement of nursing competence in these areas. Through lectures, interactive discussions, case study analysis, and application tools for patient safety, participants actively engage in learning. The program is flexible, allowing customization to meet the specific needs and goals of both the research and participants. Learning outcomes in the QSEN competency model align with Bloom’s Taxonomy, encompassing various cognitive skill levels, and focus on six competencies: patient-centered care, teamwork and collaboration, EBP, QI, safety, and informatics [23].

According to earlier research using this method [24, 25] which reported that the items used have a Cronbach’s alpha(s) ≥ 0.70 with strong recommendations of the current content.

Five nursing specialists assessed the questionnaire and endorsed its content validity. No changes were required to the chosen tool or the intervention program after the panel assessed the items, which were found to be representative of all domains of interest. With an overall Content Validity Index (CVI) of 0.88, the expert panel determined that the majority of the items were extremely relevant.

Although the CPEQ and KSAI-PCCS are validated instruments widely used to measure clinical performance and patient-centered care competencies, their specific reliability and validity have not been extensively tested in the Palestinian healthcare context. So, cultural adaptation was modified according to pilot testing. Future research should prioritize conducting context-specific validation studies to ensure the appropriateness and robustness of these tools in similar settings.

### Data collection procedure

In both the PMC and Rafidia Hospital, comprehensive approval was obtained from the Ministry of Health. Interviewed hospital administrators, which determined the objectives of the study and the expected impact of the program on nursing performance QSEN competency. A meeting was also held with nursing directors to arrange appropriate lists for filling out the questionnaire, both in Rafidia, which be without the intervention of the education program, and in the complex that will receive training on the QSEN competency program. A thoughtful process for collecting samples, taking into account the inclusion criteria. The nursing staff was then classified into the intervention group at the PMC or the control group at Rafidia Hospital. Five groups (20 nurses for each group) were set up at PMC to receive the QSEN competency program, which included pre-tests, a 4-hour QSEN competency intervention, and post-tests. At Rafidia Hospital, 64 nurses, in the control group, underwent pre- and post-questionnaire assessments with a two-week interval for the control group without the intervention program. The intervention group participated in the QSEN competency training program for four hours for each group, and homogeneity testing ensured comparability between groups. All nursing staff were informed of the objectives of the study, signed a participation agreement, and could withdraw at any time, and QSEN competency-focused training sessions began in October 2023. Following the QSEN competency intervention, final assessments of nursing competencies were conducted by redistributing the questionnaire, facilitating the assessment of the impact of the program on participants. In both groups, the pre- and post-questionnaires were numbered with the same number for the same person emphasizing the systematic nature of the study implementation.

### Ethical statements

All participants were asked for their informed consent. Participants were made aware of their freedom to leave the research at any time and without consequence. Anonymity and confidentiality were maintained by protecting the identity of the participants. Data were stored in secure, password-protected databases. Full data were given regarding the intervention duration, questionnaire completion needed from 15–20 minutes to be filled out, and reminders about the upcoming session. The institutional review board (IRB) from the University and MOH authorized the study under number (#2023/A/137/N). The researcher obtained approval to use the instruments.

### Statistical methods

Statistical Package for SocialSience (SPSS) version 28 was used to analyze the collected data. Descriptive statistics were employed to summarize and present the main features of the dataset (measures such as mean, median, mode, range, and standard deviation). Each variable of interest, including junior nurses’ KSAs, was subjected to descriptive analysis. This step allowed for a comprehensive overview of the central tendencies and variability within the dataset. Besides, reliability tests were conducted to assess the consistency and stability of the measurement instruments, particularly the questionnaire used to collect data on junior nurses’ KSAs; paired sample statistics were utilized to compare the performance of junior nurses before and after the implementation of the QSEN competency-based program. For descriptive statistics; the Shapiro-Wilk test was used to evaluate the normally distributed population of the collected data. Inferential statistics used the paired t-test of the Mann-Whitney U test if the data were normally distributed, and the Wilcoxon Signed Rank test if the data were not normally distributed.

## Result

### Participants’ characteristics

Most of the participants worked in the general surgery department (36%), about (59.5%) were females, (60.7%) were married women. There were no statistically significant differences between the two groups in these characteristics, except for their level of education and place of study. The majority of participants held a first university degree and were graduates of either An-Najah National University (25.3%) or the Ibn Sina Institute (24%), with statistically significant differences observed between the groups in these respects.

The study revealed that participants had an average age of around 28 years and an average of approximately 4 years in their current job. There was no statistically significant difference between the control and interventional groups in terms of these variables. Most variables between the two groups did not show statistically significant differences. The Mann-Whitney U test was employed to assess potential differences in quality and safety competence scores between the two groups at baseline since the data were not normally distributed Table 1.

**Table 1:** Comparison of the participants’ characteristics among control vs. interventional groups (n = 65 vs. 99 respectively).

Items	Sub-items	Total	Control (n = 65, 39.6)	Intervention (n = 99 60.4%)
			n (%)	n (%)
**Department**	Medical	32 (19.5%)	13 (20.0)	19 (19.2)
	Daily Care	17 (10.4%)	7 (10.8)	10 (10.1)
	Pediatric	23 (14.0%)	13 (20.0)	10 (10.1)
	Surgical	59 (36.0%)	19 (29.2)	40 (40.4)
	ICU	33 (20.1%)	13 (20.0)	20 (20.2)
**Gender**	Male	66 (40.5%)	28 (43.1)	38 (38.8)
	Female	97 (59.5%)	37 (56.9)	60 (61.2)
**MS**	Single	64 (39.3%)	25 (38.5)	39 (39.8)
	Married	99 (60.7%)	40 (61.5)	59 (60.2)
**Education**	BSC	137 (84.6%)	53 (81.5)	84 (86.6)
	H. Diploma	4 (2.5%)	4 (6.2)	0 (0.0)
	Master	21 (13.0%)	8 (12.3)	13 (13.4)
**University**	Ibn Sina	35 (24.0%)	17 (28.8)	18 (20.7)
	ANNU	37 (25.3%)	19 (32.2)	18 (20.7)
	Rawda	7 (4.8%)	7 (11.9)	0 (0.0)
	Andaleeb	1 (0.7%)	1 (1.7)	0 (0.0)
	Aaup	14 (9.6%)	8 (13.6)	6 (6.9)
	Alquds	24 (16.4%)	5 (8.5)	19 (21.8)
	Beitlehem	9 (6.2%)	1 (1.7)	8 (9.2)
	Almansora	1 (0.7%)	1 (1.7)	0 (0.0)
	Msu	3 (2.1%)	0 (0.0)	3 (3.4)
	Beirzeit	14 (9.6%)	0 (0.0)	14 (16.1)
	Alsaraya	1 (0.7%)	0 (0.0)	1 (1.1)
**Age**	**M = 28.6 (.12)**			
**Years of experience**	**M = 4.28 (3.37)**			
**Employment**	**M = 4.68 (3.28)**			

***Notes***: M = Mean, SD = Standard Deviation, n = number, % = frequency.

### Baseline averages level of quality and safety competence subscales among participants

Before the educational process began, it was observed that participants in the control group had higher average scores in quality and safety competence compared to those in the interventional group. Specifically, in Patient-Centered Care (17.72 ±4.87 vs. 13.99±5.24), Teamwork & Collaboration (14.93± 4.36 vs. 11.83±4.09), Evidence-Based Practice (4.10± 1.26 vs. 3.27±1.27), Quality Improvement (6.16± 2.04 vs. 4.92±1.98), Safety (13.40±3.99 vs. 10.78±4.38), and Informatics (13.89±2.83 vs. 9.92±4.05). These differences in average scores between the two groups were statistically significant (p < 0.001) and could be related to the pre-existing knowledge and experience of the participants in the control group who might have had more prior education or work experience related to quality and safety competence (Table 2).

**Table 2:** Baseline averages quality and safety competence subscales among the participants.

Quality & safety competence (Pretest)	Group	n	Mean	SD	Median	Mann Whitney U test	Z	*P* value
**Patient-Centered Care**	Control	54	17.72	4.87	19.50	1348.0	-4.027	<0.001
	Intervention	84	13.99	5.24	13.00			
**Teamwork & Collaboration**	Control	58	14.93	4.36	17.00	1431.5	-4.351	<0.001
	Intervention	86	11.83	4.09	12.00			
**Evidence-Based Practice**	Control	60	4.10	1.26	4.00	1789.0	-3.711	<0.001
	Intervention	91	3.27	1.27	3.00			
**Quality Improvement**	Control	61	6.16	2.04	6.00	1910.0	-3.675	<0.001
	Intervention	95	4.92	1.98	5.00			
**Safety**	Control	57	13.40	3.99	14.00	1788.0	-3.530	<0.001
	Intervention	95	10.78	4.38	10.00			
**Informatics**	Control	47	13.89	2.83	14.00	1001.5	-5.296	<0.001
	Intervention	93	9.92	4.05	8.00			

### The post-test averages the level of quality and safety competence subscales among the participants

Participants in the interventional group showed higher average scores in quality and safety competence subscales compared to those in the control group. Specifically, in Patient-Centered Care (22.89± 2.26 vs. 18.28±4.24), Teamwork & Collaboration (19.52± 2.36 vs. 16.57±3.72), Evidence-Based Practice (5.60±0.73 vs. 4.15±1.25), Quality Improvement (8.45± 1.08 vs. 6.48±1.81), Safety (17.12 ±1.52 vs. 13.60±3.41), and Informatics (16.82±1.99 vs. 15.06±2.31). These differences in average scores between the two groups were statistically significant (p < 0.001) (Table 3).

**Table 3:** Mann Whitney U test to assess the difference between the level of quality and safety competence subscales among the participants (Control vs. Interventional) in the study.

Quality & safety competence subscales (post)	*Hospital*	n	Mean	SD	Median	Mann Whitney U test	Z	*P* value
**Patient-Centered Care**	Control	58	18.28	4.24	19.50	766.500	-7.832	<0.001
	Intervention	94	22.89	2.26	24.00			
**Teamwork and Collaboration**	Control	56	16.57	3.72	18.00	1170.500	-5.806	<0.001
	Intervention	92	19.52	2.36	21.00			
**Evidence-Based-Practice**	Control	61	4.15	1.25	4.00	1097.000	-7.368	<0.001
	Intervention	98	5.60	0.73	6.00			
**Quality Improvement**	Control	63	6.48	1.81	7.00	1111.000	-7.412	<0.001
	Intervention	98	8.45	1.08	9.00			
**Safety**	Control	60	13.60	3.41	15.00	798.500	-7.828	<0.001
	Intervention	94	17.12	1.52	18.00			
**Informatics**	Control	51	15.06	2.31	15.00	1204.000	-5.230	<0.001
	Intervention	94	16.82	1.99	18.00			

### The difference between the baselines and posttest after intervention of average level of quality and safety competence subscales among the control group participants

The study found that among participants in the control group, the average levels of quality and safety competence subscales were nearly unchanged between the baseline (pretest) and post-test after intervention. Specifically, in Patient-Centered Care (17.68±4.78 vs. 18.14±4.37), Teamwork & Collaboration (15.13± 4.24 vs. 16.46±3.82), Evidence-Based Practice (4.17± 1.22 vs. 4.12±1.26), Quality Improvement (6.21± 2.01vs. 6.50±1.85), Safety (13.38± 3.89 vs. 13.57±3.48), and Informatics (13.89± 2.83 vs. 14.97±2.38). These differences between pretest and post-test scores after intervention among the control group participants were not statistically significant in all subscales of quality and safety competence, except for the Teamwork & Collaboration subscale, where a statistically significant increase was observed (p = 0.023). The post-test score was slightly higher compared to the baseline score (Table 4).

**Table 4:** Wilcoxon Signed Rank Test to assess the difference between the pretest and post-test after intervention of average level of quality and safety competence subscales among the interventional group participants.

Quality and safety competence subscales	Test	Mean	n	SD	Median	Z	*P* value
**Patient-Centered Care**	Pre	13.72	79	5.17	13.00	-7.460	0.000
	Post	22.77	79	2.33	24.00		
**Teamwork Collaboration**	Pre	11.81	79	4.09	12.00	-7.419	0.000
	Post	19.48	79	2.36	21.00		
**Evidence-Based Practice**	Pre	3.28	90	1.27	3.00	-7.711	0.000
	Post	5.57	90	.749	6.00		
**Quality Improvement**	Pre	4.93	94	1.98	5.00	-7.823	0.000
	Post	8.42	94	1.09	9.00		
**Safety**	Pre	10.71	90	4.38	10.00	-7.601	0.000
	Post	17.07	90	1.53	18.00		
**Informatics**	Pre	9.82	89	4.07	8.00	-7.819	0.000
	Post	16.77	89	2.03	18.00		

### Difference between the pretest and posttest after intervention of an average level of quality and safety competence subscales among the interventional group

Participants in the interventional group showed higher average levels of quality and safety competence subscales in the post-test compared to the baseline (pretest). Specifically, in Patient-Centered Care (22.77±2.33 vs. 13.72±5.17), Teamwork & Collaboration (19.48±2.36 vs. 11.81±4.09), Evidence-Based Practice (5.57± 0.75 vs. 3.28±1.27), Quality Improvement (8.42±1.09 vs. 4.93±1.98), Safety (17.07±1.53 vs. 10.71±4.38), and Informatics (16.77±2.03 vs. 9.82±4.07). However, these differences between the baseline (pretest) and post-test scores after the intervention among the interventional group participants were not statistically significant in all subscales of quality and safety competence (p > 0.05).

### Impact of QSEN on the knowledge, skills, and attitudes of junior nurses working in the Palestinian Ministry of Health

The distributions of knowledge, skills, and attitudes were found to be not normally distributed according to Kolmogorov-Smirnov and Shapiro-Wilk tests. Approximately 70% of participants in the control group had knowledge scores ranging from 29 to 79 out of 100, while in the intervention group, scores ranged from 30 to 70 out of 100. Similarly, for skills, around 70% of participants in the control group scored between 21 to 69 out of 100, compared to 26 to 60 out of 100 in the intervention group. In terms of attitudes specifically, approximately 70% of participants in the control group had scores between 24 to 77 out of 100, while in the intervention group, scores ranged from 25.5 to 66 out of 100.

The average knowledge level was slightly higher in the control group (54.06±25.06) compared to the experimental group (50.37±20.16), but this difference was not statistically significant (p = 0.29). Regarding skills and attitudes, the average scores in these areas were also slightly higher in the control group (skills, 45.1±23.99, attitudes, 50.67±26.57) compared to the intervention group (skills, 43.77±17.73, attitudes, 45.88±23.99). However, these differences were not statistically significant (p > 0.05). Therefore, both groups had similar average levels of skills and attitudes before the educational interventions. These findings suggest that there was no significant disparity between the groups before the educational procedures, allowing for a meaningful comparison of the impact of the interventions.

To assess the impact of an educational procedure on quality and safety knowledge, skills, and attitudes among participants in the intervention group, a Mann-Whitney U test was employed due to non-normal distribution. Table 3 confirms that post-procedure, participants in the intervention group exhibited significantly higher levels of knowledge compared to those in the control group (77.02±16.39 vs. 49.19±26.91, p < 0.001). Similarly, post-procedure, participants in the intervention group also demonstrated significantly higher levels of quality and safety skills (70.16±13.91 vs. 44.61±25.13, p < 0.001) and attitudes (75.47 ±13.94 vs. 46.16± 27.41, p < 0.001) compared to the control group. These findings indicate a substantial positive impact of the educational intervention on knowledge, skills, and attitudes related to quality and safety.

The Wilcoxon Signed Rank Test was used to examine the difference in average levels of quality and safety knowledge, skills, and attitudes between baseline (pretest) and posttest among participants in the control group of the study since the data was not normally distributed. The analysis revealed that there was minimal change in the average levels of quality and safety knowledge (54.06 ±25.06 vs. 49.19±26.91), skills (45.10± 23.99 vs. 44.61±25.13), and attitudes (50.67± 26.57 vs. 46.16±27.41) from baseline to posttest among participants in the control group. These differences were not statistically significant (p > 0.05), indicating that the intervention did not lead to measurable improvements in these areas among the control group.

The study utilized the Wilcoxon Signed Rank Test to assess changes in average levels of quality and safety knowledge, skills, and attitudes from pretest to post-test among participants in the interventional group since the data was not normally distributed. Results indicate significant improvements in quality and safety knowledge (50.37±20.16 to 77.02±16.39), skills (43.77±17.73 to 70.16±13.91), and attitudes (45.89±20.22 to 75.47±13.94) following the intervention compared to baseline measures. These differences were statistically significant (p < 0.05), highlighting the effectiveness of the intervention in enhancing participants’ understanding and capabilities in quality and safety practices Table 5.

**Table 5:** Wilcoxon Signed Rank Test to assess the difference between the pretest and post-test after intervention of average level of quality and safety knowledge, skills, and attitude among the interventional group participants in the study.

Quality & safety		N	Mean	SD	Median	Z	*P* value
**knowledge**	Pre	83	50.37	20.16	49.00	-6.551	0.000
	Post	91	77.02	16.39	77.00		
**Skills**	Pre	87	43.77	17.73	40.00	-7.084	0.000
	Post	93	70.16	13.91	74.00		
**Attitudes**	Pre	88	45.89	20.22	43.00	-7.358	0.000
	Post	94	75.47	13.94	78.00		

Wilcoxon Signed Rank Test.

## Discussion

The categories of QSEN are related to critical areas of learning of the knowledge, skills, and attitudes learned during the clinical practice of junior nurses. The implementation of the QSEN program resulted in a marked increase in junior nurses’ knowledge, skills, and attitudes toward patient safety protocols. The study revealed significant scores in quality and safety competence compared to those in the interventional group across Patient-Centered Care, Teamwork & Collaboration, Evidence-Based Practice, Quality Improvement, Safety, and Informatics that were observed in all scores. This difference could potentially be attributed to a higher proportion of participants with diplomas and master’s degrees in the control group compared to the interventional group. Additionally, challenges such as heavy workloads and nurse shortages may have limited participation in patient safety training courses among nurses in both groups. Potential confounding factors like prior education, clinical experience, and workplace support were identified.

Although the QSEN program showed notable increases in attitudes and knowledge, the comparatively modest skill gains point to possible obstacles to learning new abilities. This disparity is consistent with earlier studies that highlight the difficulties in applying theoretical understanding to clinical practice, especially in environments with low resources. Opportunities for experiential learning may have been hampered in Palestine by structural issues such resource poverty, high patient-to-nurse ratios, and restricted access to simulation facilities. Furthermore, the form of the QSEN curriculum may provide more weight to the development of cognitive and emotive domains than psychomotor skills. Future implementations could close this gap if these issues are addressed with improved clinical training opportunities and institutional support. The impact of the health sector on junior nurses’ attitudes, abilities, and knowledge. Cengiz and Yoder [8] found that among various QSEN fundamental skills, collaboration and teamwork had the highest average scores across both study groups. They also noted that registered nurses in managerial roles scored the lowest scores in Evidence-Based Practice (EBP), while recently hired nurses showed the least proficiency in informatics. These findings align with Bertch [26] who reported that newly employed registered nurses scored an average of (M = 67.6%, *SD* = 10.32) on knowledge assessments with scores ranging from 47% to 84%. Registered nurses in staff leadership positions scored slightly higher, averaging (M = 72.1%, *SD* = 8.06), ranging between 50% and 88%. Combining scores from staff leadership and newly hired nurses resulted in an average score of (M = 69.2%, *SD* = 9.76). In contrast, the present study’s findings diverged from Kakemam, Albelbeisi [27] who found that nurses rated their overall patient safety competency at 3.44 out of 5.0, with 41% rating their competency below 3.0, indicating insufficient PS proficiency. Kakemam, Albelbeisi [27] also noted that nearly 60% of nurses lacked adequate knowledge in PS-related areas, potentially due to a lack of formal university education focusing on patient safety concerns among working nurses.

The study looked at each QSEN core competency to determine how the two groups’ quality and safety competence scores differed once the post-intervention education process started. It was discovered that individuals in the interventional group outperformed those in the control group in terms of average scores in the quality and safety competence subscales, with statistically significant differences (p < 0.001) noted in all subscales. Junior nurses’ self-reported assessments, in which they characterized themselves as "novice/familiar" in using graphical tools for QI data visualization and basic statistical analysis, corroborated the lower results in the Quality Improvement (QI) area. These findings align with Bertch [26], who found that RNs’in staff leadership positions scored highest on knowledge assessments related to teamwork and collaboration (81.1%) and informatics (80.9%). In contrast, their performance was lower in quality improvement (QI) knowledge (70.8%) and evidence-based practice (EBP) knowledge (62.4%). Bertch [26] also observed that self-reported skills evaluations among these nurses confirmed their weaker proficiency in the QI domain.

The study revealed a significant difference in the average levels of all quality and safety competence subscales between the control and intervention groups. Participants in the Intervention group demonstrated higher average levels of quality and safety competence compared to those in the Control group. Conversely, among participants in the Control group, who did not undergo any educational intervention, average levels of quality and safety competence subscales remained nearly unchanged between the pre-test and post-test assessments. Among participants in the control group of the study, changes in average levels of quality and safety competence subscales from baseline to post-test after intervention showed no statistically significant differences (p > 0.05), except for the "Teamwork Collaboration" subscale, where a slight increase was observed. Additionally, the control group displayed slightly higher average skills and attitudes compared to the intervention group. Specifically, the control group’s average scores were 45.1 and 50.67 out of 100 for skills and attitudes, respectively, whereas the intervention group averaged 43.77 and 45.88 out of 100 for skills and attitudes, respectively. This difference might be attributed to the control group’s perceived higher competence level, possibly due to their longer years of experience.

Bertch [26] concluded that years of experience as a registered nurse (RN) did not affect understanding of attitudes, knowledge, and skills related to QSEN core competencies among newly hired RNs or those in staff leadership roles. In contrast, the current study demonstrated significant improvements in all subscales of quality and safety competence among participants in the intervention group. Following a comprehensive educational program, these participants showed statistically higher average levels of quality and safety competence in the post-test compared to baseline assessments. This underscores the effectiveness of the educational intervention in enhancing participants’ knowledge and skills in these areas.

In terms of baseline knowledge distribution, approximately 70% of participants in the control group scored between 29 to 79, while in the intervention group, scores ranged from 30 to 70. This suggests that a majority of respondents lack mastery of the skills necessary to apply QSEN core competencies effectively in practical scenarios. This gap indicates a widespread deficiency in understanding QSEN critical competencies across various experience levels.

Maxwell [28] did not establish a specific passing score for QSEN core competencies, leaving the determination of competency levels ambiguous. However, conventionally, a score of 70% is often considered a benchmark for competence or an acceptable passing grade. This suggests that achieving a score of 70% on the knowledge section of the questionnaire could be considered indicative of QSEN competency.

The study demonstrated that participants in the procedural group exhibited significantly higher average levels of knowledge, skills, and attitudes (KSAs) compared to those in the control group after the educational intervention. Among individuals in the control group, there was no notable change in the median amount of safety and effectiveness KSAs between the pre-test and post-test phases. Conversely, participants in the interventional group showed a significant increase in quality and safety KSAs from the pre-test to the post-test. These findings suggest that higher levels of competence in quality and safety skills, encompassing knowledge and attitudes, correspond to greater awareness of patient safety (PS) and adverse events among nurses. Moreover, nurses with enhanced skills and attitudes are more likely to report adverse events to their managers or PS departments.

Similarly, Bell-Wilson [7] explored the integration of QSEN competencies in nursing curricula, finding that a significant percentage of programs include information on QSEN skills. However, their study revealed that nursing programs offer fewer courses specifically dedicated to these competencies. Pre-licensure programs were found to prioritize patient safety, teamwork, and collaboration in their curricula. Faculty and academic leaders reported using diverse teaching methods to impart these competencies but highlighted gaps in their knowledge of quality enhancement and evidence-based practice (EBP). They noted a correlation between their reported lack of expertise and lower levels of student competency in these areas.

Altmiller and Pepe [29] discovered that 47% of participants had jobs in clinical settings in addition to their academic employment, suggesting that faculty education may have an impact on QSEN skills in real-world practice settings. Teachers can be extremely helpful in assisting nurses in incorporating QSEN principles into their clinical practice, even if they obtained their degrees before to the establishment of these skills.. By acting as mentors and role models in clinical settings, nurse faculty members can significantly impact both seasoned and newly graduated registered nurses. The study also indicated that a majority of respondents were actively involved in curriculum development.

However, the training program effectively increased nurses’ safety ratings and knowledge, indicating that the instructional approach alone heightened awareness of quality and safety competencies. Nevertheless, there was an unexpected outcome: scores in the skills competency area notably decreased for the control group. Shepard, Spencer [30] reported no significant improvement in cognition and found participants had insufficient comprehension ratings following the simulation. Goekcimen, Schwendimann [31] similarly observed no significant increase in student scores on tests assessing procedural understanding in nursing, though improvement was noted in other categories.

Burt and Corbridge [32] investigated the effectiveness of simulations in enhancing knowledge and satisfaction. They found that while knowledge assessments were more extensive, there were no significant differences observed between the two subgroups. However, the outcomes of the knowledge test were deemed suboptimal. Cho, Lee [33] conducted a study focusing on therapeutic categories and found significant improvements in students’ understanding of patient safety, their skills, and their attitudes toward quality and safety competence. Their findings indicate that a competency-based program for enhancing quality and safety education successfully improves these aspects among students.

Kirwan and Riklikiene [34] discovered that patient safety (PS) is integrated into nursing training across twenty-seven countries, but its incorporation is less prevalent in European Union nations. They noted that regulations linking PS and quality are largely absent globally, except in the United States. Steven, Tella [35] demonstrated that international collaboration can hasten progress by sharing experiences from PS-related educational events. These findings suggest several interpretations. Firstly, the study employed a comprehensive approach to design its knowledge assessment, focusing on the quality of care scenarios provided to each student. Each scenario was pivotal in assessing students’ grasp of general safety and high-quality care principles, rather than specific case details.

The study highlights that nurses’ confidence in demonstrating quality and safety competencies can be enhanced through education. Confidence in these skills is crucial in nursing, a profession heavily focused on clinical practice. The research underscores the importance of carefully planning and implementing assessment techniques to effectively evaluate educational outcomes. It also cautions against assuming nurses will acquire necessary knowledge independently, emphasizing the need for comprehensive teaching. Furthermore, the university is actively involved in advancing QSEN competency-related initiatives. As faculty members increasingly integrate QSEN content into curricula, more nursing graduates are expected to possess robust knowledge and practical experience in these competencies. Their proactive role is pivotal in driving this educational transformation [36].

Current research identifies a gap in understanding how pre-licensure nursing students perceive QSEN skills, as most studies focus on assessment rather than student perspectives Cengiz and Yoder [8, 37]. Notably, studies on associate degree education and faculty viewpoints were excluded from the literature review. Understanding these perceptions could guide modifications in instructional approaches to foster a culture of high-quality, safety-oriented nursing. Bridging academia with practice is crucial for advancing the field, and initiatives like the QSAAN project and the QSEN program offer numerous opportunities for collaboration and growth [38].

Nursing partnerships focused solely on improving the quality and safety environment have the potential to significantly influence patient care quality and safety across healthcare settings and in shaping future health policies [39]. Equipping healthcare providers with essential tools to enhance their knowledge and skills is crucial for maintaining a safe and high-quality work environment. Therefore, the Quality and Safety Education for Nurses (QSEN) initiative aims to provide nurses with the necessary information, skills, and attitudes to enhance healthcare quality and security, particularly within the Palestinian Ministry of Health. The national QSEN project encourages nurses to reconsider their approach to nursing care delivery, focusing on ensuring quality and safety. By identifying and addressing gaps between current practices and ideal standards, QSEN empowers nurses to improve healthcare outcomes effectively.

### Implications for research and practice

This study has several recommendations that focus on the urgent need for deliberate integration of QSEN core competencies into local and regional nursing school curricula, along with validation of these skills for hospital administration. Furthermore, it is recommended that the Ministry of Health (MOH) should formalize the QSEN competency assessment as an ongoing part of every registered nurse’s professional development plan. Allocate resources towards the evaluation and development of nurse preceptors to enhance their understanding and demonstration of QSEN competencies and KSAs. Further research is necessary to explore specific interventions in educational QSEN competency programs within hospitals, targeting improvements in nurses’ patient-centered care competencies, as assessed by the KSAI-PCCS.

To sum up, the QSEN program has significantly enhanced the knowledge, skills, and attitudes of junior nurses at the Palestinian Ministry of Health, contributing to improved patient care. Continued investment in QSEN education and its principles is crucial for sustaining and advancing healthcare quality and safety. Future research should focus on long-term impacts and broader applications to further validate and enhance the program’s effectiveness.

### Strengths and limitations

This study encapsulates a pioneering issue conducted in Palestine, assessing the effects of a QSEN-based program on the knowledge, skills, and attitudes (KSAs) of junior nurses employed by the Palestinian Ministry of Health. While the research yielded significant results, several limitations were noted. The current study’s findings are subject to limitations that warrant careful interpretation. The variability in baseline competencies of knowledge, skills, and attitudes among junior nurses likely influenced the degree of improvement observed, with potential ceiling effects in high-performing participants and amplified gains in those with lower initial competencies. Additionally, the small sample size may have limited the statistical power to detect significant effects and reduced the generalizability of the findings to the wider population of junior nurses in Palestine. Additionally, the reliability and validity of instruments were described. However, the reliability and validity of these tools in the Palestinian context were not extensively tested, which is a limitation. To address these issues, future research should employ stratified analyses, covariate adjustments, and larger, multicenter samples to enhance the robustness and applicability of the results.

## Conclusions

This study demonstrates that the educational intervention raised junior nurses’ KSAs for six QSEN competencies. The QSEN program has significantly enhanced the knowledge, skills, and attitudes of junior nurses at the Palestinian Ministry of Health, contributing to improved patient care. Continued investment in QSEN education and its principles is crucial for sustaining and advancing healthcare quality and safety. Future research should focus on long-term impacts and broader applications to further validate and enhance the program’s effectiveness. Research indicates that when healthcare organizations apply QSEN principles, they often see increases in patient safety, staff satisfaction, and a decrease in medical errors. Thus, prioritizing QSEN integration raises safety and quality standards in healthcare in general while also producing qualified nurses.

## Supporting information

S1 Checklist TREND statement checklist. (DOC)

S1 Dataset (XLSX)

S1 File Course education (duration 4 hours). (DOCX)
