# Supplementary material for: The impact of The Quality and Safety Education (QSEN) program on the knowledge, skills, and attitudes of junior nurses
Source: PLoS One. 2025 Jan 24;20(1):e0317448. doi: 10.1371/journal.pone.0317448 (PMC11761152; doi:10.1371/journal.pone.0317448)
Supplement: S1 File — (DOCX) [file pone.0317448.s003.docx]

Course education (duration 4 hours)

| **Topic of QSEN competency education program** | **Method of learning** | **Time** |
| --- | --- | --- |
| Session 1: Introduction to the QSEN competency program  Brief Introduction to the QSEN Competency Program Importance of Quality and Safety in Nursing. The vital role of nurses in healthcare. Ensuring Safe and High-Quality Patient Care.  Introduction to Quality and Safety. Discussion of the impact of quality and safety on patient outcomes.  Patient safety. | Presentation &discussion | (10minutes) |
| Session 2: The purpose of Quality and QSEN Competency. Important of QSEN Competency  How QSEN Competency influences my practice, Staff problem-solve as a team, Staff focus on unit goals collectively, Staff reach an intervention and put it into practice. | Presentation &discussion | 10 minutes |
| Overview of PS and quality of care of  QSEN program, Quality and safety in high–reliability organization PS  Benchmarking quality performance  Tools of quality improvement  The future role of registered nurses in PS and quality | Presentation &discussion | 10 minutes |
| Session 3: Development of the QSEN competency program, QSEN competency was created in 2005 by Linda Cronenwett, PhD, RN, FAAN, and a team of quality and safety professionals (Disch, 2012). The Robert Wood Johnson Foundation, the nation's biggest charity devoted to enhancing health care, then provided funding for QSEN competency (QSEN, 2012).  • Every one of their six skills is a learning target for nursing graduates and pre-licensure students (QSEN, 2012). | Presentation &discussion | 5minites |
| Session4: Models of QSEN Competency  Model one  PCC: definition, understanding the importance of PCC. What can the nurses do as individuals to help meet the PCC goals? Patients’ values, religion, culture, and individual needs must be assessed. Communication with other caregivers regarding individual patient needs. Allow the family and the patient time to ask questions, time to visit with family, and provide emotional support as needed. Recognize the patient or designee as the source of control and full partner in providing compassionate and coordinated care based on respect for patient's preferences, values, and needs” (QSEN, 2012). Provide information on medications and disease processes to keep the patient informed.  Keep patient informed on scheduled procedures, blood draws, and testing that is to be done. | -Presentation 5 minutes.  -Scenario for Patient interactions 10 minutes.  - Discussion (10 minutes).  - 4. Conclusion (5 minutes) | 30 minutes |
| Models tow: Teamwork Collaboration: Function effectively within nursing and inter-professional teams, fostering open communication, mutual respect, and shared decision-making to achieve quality patient care” (QSEN, 2012).  The significance of interdisciplinary teamwork in healthcare. Effective communication within the healthcare team. Encourage participants to reflect on their experiences. Teach the nurses how to use the KSAs needed for teamwork and collaboration to improve quality and safety in their practice as nursing professionals (Disch, 2012). | Introduction presentation (5 minutes)  Scenario Assignment (5 minutes):  Simulation (10 minutes):  Discussion (5minutes)  Conclusion (5 minutes) | 25 minutes |
| Model three EBP is the integration of clinical expertise, patient values, and the best research evidence into the decision-making process for patient care. The evidence alone does not decide for you; however, it can help support the patient care process. The full collaboration of these three components into clinical decisions improves the opportunity for optimal clinical outcomes and quality of life. EBP requires new skills of the health care professional, including effective literature searching and the use of formal rules of evidence in evaluating the literature. | introduction (5minute)  Study Presentation (10 minutes)  Evidence Application (10 minutes)  Discussion (5minutes)  Conclusion (5 minutes):  Clinical Question:  Search for Evidence: two articles.  Application of Evidence from Articles.  Evaluation of Outcomes | 25minutes |
| Model Four: QI  Definition: “Use data to monitor the outcomes of care processes and use improvement methods to design and test changes to continuously improve the quality and safety of health care systems.” (QSEN, 2012). QSEN competency standards for QI give healthcare professionals a rule of measurement against which we can judge our acquirement of knowledge and how we choose to put that knowledge to use  Tools of quality improvement | Presentation 5 minutes.  -Scenario for Patient interactions 10 minutes.  - Discussion (10 minutes).  - 4. Conclusion (5 minutes) | 30 minutes |
| Models five Safety  Definition: “Minimizes risk of harm to patients and providers through both system effectiveness and individual performance” (QSEN, 2012). Safety precautions go beyond the six rights of medication administration, fall precautions, and call lights within reach.  Discuss safety practices, potential risks, and hazards in healthcare settings. Present techniques for error prevention and safety promotion. Are nurses encouraged to share their mistakes or near misses, or are they afraid of getting reprimanded? Nurses should be encouraged to share openly about errors because everyone can learn and improve from that mistake (Durham, & Sherwood, 2008).  Provide a case study that involves adverse events or near misses, and SSC, Asks participants to analyze the case study and suggest actions to prevent similar incidents.  Q&A and Summary. | Presentation 5 minutes.  -Scenario for Patient interactions 10 minutes.  -Group Discussion (10 minutes).  - 4. Conclusion (5 minutes) | 30 minutes |
| Models six Informatics.  Definition: "Use information and technology to communicate, manage knowledge, mitigate error, and support decision making” (QSEN, 2012). Bedside computer charting and barcode scanning can assist in keeping information up to date and in real time (Durham, & Sherwood, 2008).  The module explores how innovations in health information technology have changed our work with nursing students in classroom, clinical, and lab settings. Strategies to integrate informatics content across these settings are suggested, along with key resources for further information.  Open the floor for questions and answers. Summarize key points and takeaways from the sessions.  Assessment and Feedback (15 minutes)  Collect feedback on the program content, delivery, and any areas that may need improvement. | Introduction (5 minutes)  Overview of Nursing Informatics(5minutes)  Case Study Presentation (10minutes)  Case Study: Medication Errors  Discussion (10 minutes)  Conclusion (5 minutes) | 30 minutes |
| Session7: Examples of PS forms:  •PS Incidence Report  •Root cause analysis  •Morse scale  •SSC  •Effective communication (SBAR)   - PDSA | Activity Forms filling | 30 min  Each form took 5 mint |
